# A genomic footprint of hybrid zone movement in crested newts

**DOI:** 10.1002/evl3.9

**Published:** 2017-05-09

**Authors:** Ben Wielstra, Terry Burke, Roger K. Butlin, Aziz Avcı, Nazan Üzüm, Emin Bozkurt, Kurtuluş Olgun, Jan W. Arntzen

**Affiliations:** ^1^ Department of Animal and Plant Sciences University of Sheffield S10 2TN Sheffield United Kingdom; ^2^ Naturalis Biodiversity Center 2300 RA Leiden The Netherlands; ^3^ Department of Ecology and Evolutionary Biology University of California Los Angeles California 90095; ^4^ Department of Marine Sciences University of Gothenburg S 405 30 Gothenburg Sweden; ^5^ Department of Biology, Faculty of Arts and Sciences Adnan Menderes University 09010 Aydın Turkey

**Keywords:** Hybridization, introgression, phylogeography, secondary contact, speciation, species displacement, *Triturus*

## Abstract

Speciation typically involves a stage in which species can still exchange genetic material. Interspecific gene flow is facilitated by the hybrid zones that such species establish upon secondary contact. If one member of a hybridizing species pair displaces the other, their hybrid zone would move across the landscape. Although theory predicts that moving hybrid zones quickly stagnate, hybrid zones tracked over one or a few decades do not always follow such a limitation. This suggests that hybrid zones have the potential to traverse considerable distances over extended periods of time. When hybrid zones move, introgression is predicted to result in biased gene flow of selectively neutral alleles, from the receding species into the advancing species. We test for such a genomic footprint of hybrid zone movement in a pair of crested newt species (genus *Triturus*) for which we have a priori support for westward hybrid zone movement. We perform a multilocus phylogeographical survey and conduct Bayesian clustering analysis, estimation of ancestry and heterozygosity, and geographical cline analysis. In a 600 km wide area east of the present day hybrid zone a genomic footprint constitutes empirical evidence consistent with westward hybrid zone movement. The crested newt case suggests that hybrid zone movement can occur over an extensive span of time and space. Inferring hybrid zone movement provides fundamental insight into historical biogeography and the speciation process, and we anticipate that hybrid zones will prove to be far more mobile than currently appreciated.

**Impact Summary**

Where recently diverged species meet in nature they often establish zones in which they hybridize and exchange genetic material. When one such species then displaces the other, their hybrid zone is, in consequence, moving. However, there is disagreement about how far a hybrid zone can travel before its position stabilizes. A key prediction of hybrid zone movement is that the receding species leaves behind a trail of selectively neutral alleles within the expanding species. Such a genomic footprint would document the hybrid zone's dynamic history. Using this rationale, we screen dozens of genes in crested newts. The strongly asymmetrical and geographically extensive introgression we discover supports hybrid zone movement and suggests that such movement can proceed over considerable time and space. We anticipate that hybrid zone movement occurs more frequently than currently appreciated and argue that it facilitates the identification of the genomic regions driving speciation.

## Introduction

Speciation typically involves the gradual build‐up of genetic incompatibility between diverging gene pools (Wu and Ting 2004). The corollary is a stage during the speciation process where reproductive isolation is sufficient for species to maintain their overall integrity while they are still capable of producing hybrids with nonzero fitness, providing a window of opportunity for alleles to be exchanged between species (Mallet 2005; Abbott et al. 2013). Hybrid zones, where species meet and admix in nature, are key to understanding introgression (Barton and Hewitt 1985; Hewitt 1988); steep clines for those alleles that underperform in the foreign species align with (and in effect define) the species boundary (Barton 2001), while positively selected alleles can permeate the foreign species’ range (Barton 2001; Arnold and Martin 2009), as can neutral ones, albeit with some delay due to the barrier effect of the zone (Barton and Hewitt 1985; Currat et al. 2008).

Hybrid zones are often positioned at an environmental transition (Endler 1977), suggesting that the species involved are adapted to different environments. A hybrid zone established upon secondary contact may not be at an equilibrium position, based on the competitive abilities of the species involved, or that equilibrium position may shift in response to climate change. Under such circumstances, exogenous selection is expected to promote the replacement of one species by the other (Arntzen 1978; Taylor et al. 2015). This is equivalent to an asymmetry in fitness at many loci across the genome, with hybrid zone movement as a consequence. Such movement has occasionally been observed for hybrid zones tracked over one or a few decades (Arntzen and Wallis 1991; Buggs 2007; Taylor et al. 2014; Leaché et al. 2017). It follows that hybrid zones potentially traverse considerable distances, over extended periods of time. Yet, tension zone theory predicts hybrid zones are easily trapped at environmental density troughs (Buggs 2007; Abbott et al. 2013). In fact, empirical evidence for hybrid zone movement over a time frame of hundreds to thousands of years is very limited and often inconclusive (Shaw et al. 1993; Wang et al. 2011; Wang et al. 2014).

Patterns of introgression across a hybrid zone could be exploited to test its long‐term mobility. A geographical asymmetry in introgression may reflect a selectively neutral allele that was left in the wake of a moving hybrid zone (Buggs 2007) or an advantageous allele that was pulled across a stable hybrid zone into the foreign species by positive selection (Ballard and Whitlock 2004). Based on a single marker, distinguishing between these two competing hypotheses is not straightforward (Dasmahapatra et al. 2002; Toews and Brelsford 2012). Yet, at the scale of the genome, the magnitude, direction, spatial concordance, and detectability of asymmetric introgression are expected to differ (Barton and Hewitt 1985). Universally advantageous alleles flow independently across a hybrid zone into either species with little delay (Barton 2001). This process is difficult to detect in natural hybrid zones, where favored alleles likely had ample time available to spread into the foreign species and reach fixation (Hewitt 1988). However, examples are known from recent, anthropogenically induced hybrid zones (Fitzpatrick et al. 2010). More restricted asymmetric gene flow may result from genetic incompatibilities and cause displacement of some clines by distances comparable to the cline widths (Payseur et al. 2004). In either case, the direction of displacement is likely to be locus, or possibly chromosome specific.

Moving hybrid zones leave a different spatial signature. In the area of species displacement the introgression of neutral alleles is exaggerated from the retreating to the advancing species and, because this introgression is only subject to drift, it will be geographically stable on an evolutionary time scale (Barton and Hewitt 1985; Currat et al. 2008). Clines for individual loci may be displaced far from the zone centre. Displaced loci may be common, in contrast to predictions from asymmetrical selection on individual loci. Hence, genomic footprints of hybrid zone movement should be readily detectable in nature, given data from a sufficiently large sample of loci. Predictions are straightforward: evidence of genetic admixture, introgression of species‐diagnostic alleles, and the displacement of geographical clines, should be biased to one side of the hybrid zone.

**Figure 1:**
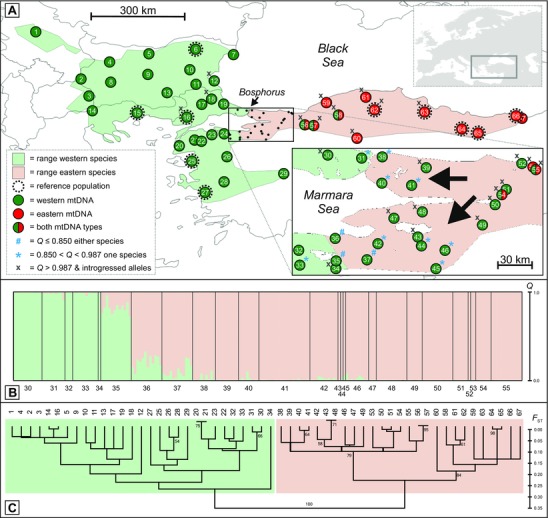
Sampling and genetic distinction of two crested newt species. (A) Background color reflects approximate species ranges based on nuclear DNA. Localities are represented by numbered dots, with colors reflecting mtDNA type and additional symbols (#, *, and x) reflecting different degrees of nuclear genetic admixture as identified by Structure. (B) Structure plot showing *Q* scores for the individuals from localities 30–55, indicating genetic contributions from the western or eastern species. Note that Structure did not suggest any admixture in the remaining localities (1–29 and 56–67). (C) Neighbor‐Joining tree for localities constructed from the matrix of pairwise corrected *F*_ST_ distance from nuclear DNA, rooted by midpoint rooting, with support values based on 1000 bootstrap replicates (only values > 50% shown).

Hybrid zone movement has previously been suggested to explain extensive, unidirectional mtDNA introgression between two hybridizing crested newt species, distributed in Asiatic Turkey and the south‐eastern Balkan Peninsula (Fig. 1A). The eastern species, *Triturus anatolicus*, is thought to have expanded at the expense of the western species, *T. ivanbureschi*, after seismic activity caused the marine waterway connecting the Black and Marmara Seas to reroute westwards (Wielstra et al. 2013a). These two recently recognized species are genetically distinct, but are as yet not known to differ in morphology, ecology, or life history (Wielstra and Arntzen 2016). To test the hypothesis of long‐term movement of the crested newt hybrid zone, we determine the degree and direction of introgression for 49 nuclear markers.

## Methods

We here outline our methods and refer to the Supplementary Methods for further details. Based on published protocols (Wielstra et al. 2013b; Wielstra et al. 2014) we sequenced one mitochondrial and 49 nuclear DNA markers, for 428 crested newt individuals from 67 localities (Fig. 1; Table S1). Per species we selected three individuals from five localities, positioned away from the putative hybrid zone, which we considered unaffected by interspecific gene flow. MtDNA haplotypes were assigned to species by Bayesian phylogenetic reconstruction in MrBayes 3.2.2 (Ronquist et al. 2012), with a model of sequence evolution chosen with jmodeltest 2.1.7 (Darriba et al. 2012). Genetic divergence between the mtDNA clades representing the two species was determined with DNAsp 5 (Librado and Rozas 2009). Using the full nuclear DNA dataset we determined genetic admixture with Structure 2.3.3 (Pritchard et al. 2000) and determined the most likely number of gene pools based on the Δ*k* criterion (Evanno et al. 2005) as implemented in CLUMPAK (Kopelman et al. 2015). We calculated multilocus genetic divergence between species in GENEPOP (Rousset 2008) and constructed a population tree in POPTREE 2 (Takezaki et al. 2010). Based on 12 fully and six nearly diagnostic nuclear DNA markers we determined individuals’ genetic composition with HIest (Fitzpatrick 2012). We conducted geographical cline analysis with HZAR (Derryberry et al. 2014). We determined whether clines, fitted to each of the (nearly) diagnostic markers and the mtDNA marker, were displaced in relation to a cline fitted to the Structure *Q* score based on all 49 nuclear markers, by comparing both the two log‐likelihood unit support limits for the cline center of the chosen model and that of the Structure cline, and on the difference in AICc score between the chosen model and that same model but with the cline center refitted to that of the Structure cline. Finally, we estimated effective selection, using life‐time dispersal distance inferred from admixture linkage disequilibrium (LD) from the hybrid index based on the nondisplaced (nearly) diagnostic nuclear DNA markers, and compared the observed cline width with that expected under neutrality, following Barton and Gale (1993). To estimate recombination rate we took information on the average number of chiasmata observed in another crested newt species, *T. carnifex*, from Wallace et al. (1997). Based on estimates of the age of maturity for both species (Üzüm 2006), we set generation time to 3.5 years. The closing of the Izmit Gulf – Lake Sapanca – Sakarya Valley waterway, thought to have initiated secondary contact, corresponds to the onset of the Holocene at c. 12 Ka (Elmas 2003).

## Results and Discussion

Bayesian clustering analysis with Structure partitions individual crested newts into two parapatrically distributed groups, corresponding to the western and eastern species (Fig. 1B; Table S1). The multilocus pairwise *F*_ST_ distance between the two species is 0.49 and a population tree confirms they form two distinct genetic clusters (Fig. 1C). In a few localities we observe individuals with considerable genetic admixture (arbitrarily defined as a Structure *Q* score < 0.850 of belonging to either species), pinpointing the position of the hybrid zone to the narrow strip of the distribution south of the Marmara Sea (localities 35–37 in Fig. 1A).

**Figure 2:**
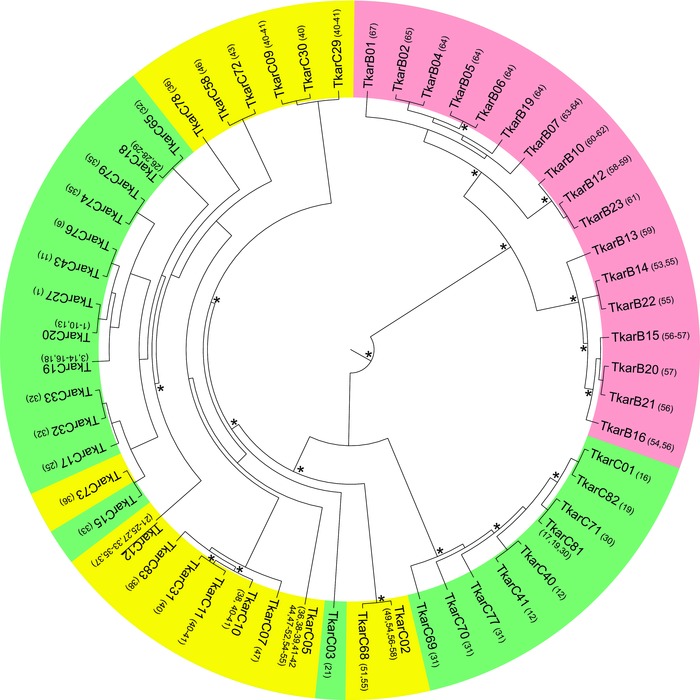
Bayesian mtDNA phylogeny for two crested newt species. Haplotypes of the western (TkarC) and eastern (TkarB) species are shown on green and red backgrounds, respectively, while western haplotypes introgressed into the eastern species are shown on a yellow background (with one underlined haplotype found in both species). Numbers in parentheses following haplotype labels correspond to localities in Fig. 1. The outgroup is not shown. Asterisks reflect posterior probability values over 0.95 (lower values are not shown).

The distribution of the two distinct (D_XY_ = 0.055) mtDNA clades (reciprocally monophyletic with a posterior probability of 1.0) representing the two species (Fig. 2; Table S2) does not fully coincide with their ranges as based on the nuclear genome (i.e., the Structure results). The mismatch roughly spans the western third of the range of the eastern species, over c. 350 km (localities 36–58 in Fig. 1A). The introgression of multiple distinct and geographically structured western mtDNA clades into the eastern species (Fig. 2) supports the hybrid zone movement hypothesis; under positive selection introgressed haplotypes are expected to be identical or similar to haplotypes found in the hybrid zone today. The mtDNA data delineate the minimum “displacement zone” over which we hypothesize the hybrid zone has shifted, and hence where we predict a genomic footprint for nuclear markers.

We first consider whether the geographical distribution of Structure *Q* scores fits the expectation of asymmetrical introgression of nuclear markers between the two species. Guided by reference individuals from localities positioned away from the hybrid zone (see Fig. 1A), the threshold to qualify as pure eastern or western species is determined as a Structure *Q* score ≥ 0.987 for the respective species; individuals with a lower score are considered admixed. Signs of minor genetic admixture (defined as belonging to a species with 0.850 < *Q* < 0.987) are limited to the vicinity of the hybrid zone in the western species (localities 31 and 33 in Fig. 1A), while they span a more extensive area in the eastern species (localities 38, 40–42, and 44–46). This asymmetry is reflected in the Structure plot by the “tail” of western genotypes in the eastern species (Fig. 1B).

**Figure 3:**
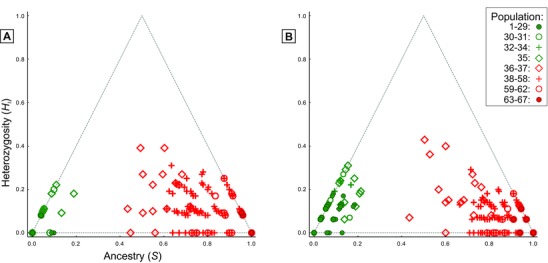
Asymmetric introgression between two crested newt species. (A) Heterozygosity (the fraction of markers heterozygous for alleles from both species) versus ancestry (the fraction of alleles derived from each species) plot based on 12 diagnostic markers. (B) The same plot with six nearly diagnostic markers added. The left corner of the triangle corresponds to a purely western genotype, the right corner to a purely eastern genotype, and the upper corner to an F1 hybrid.

Next, we determine the introgression pattern of 12 diagnostic nuclear markers (with fixed allelic differences in the reference individuals for both species). Western alleles are found in the eastern species in a much more extensive area (localities 39, 43, and 47–63 in Fig. 1A) than the reverse (localities 12, 16, 17, 30, and 34), reaching up to c. 600 km east and c. 200 km west of the hybrid zone. Furthermore, the number of alleles involved differs distinctly. Plots of heterozygosity against ancestry based on the 12 diagnostic and six nearly diagnostic nuclear markers (with a frequency difference > 0.80) show that there are far more predominantly eastern individuals that possess some western alleles than the reverse, that is the individuals plotted in Fig. 3 are more widely spread in the right corner, indicating introgression, than in the left corner, where they are concentrated close to the “pure” genotype.

**Figure 4:**
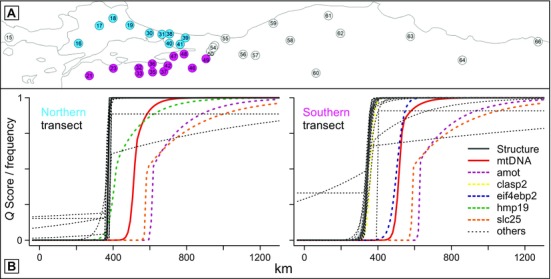
Geographical cline analysis for two crested newt transects. (A) Localities (numbered as in Fig. 1) are arranged along two transects running either north (blue, across the Bosphorus) or south (pink, through the hybrid zone) of the Marmara Sea, with a shared western starting point and eastern section (white localities). (B) Geographical cline fits along the northern and southern transect for the Structure *Q* score (heavy dark gray line with the narrow 95% credible cline region shaded light gray) and the individual mtDNA and (nearly) diagnostic markers (thin black interrupted lines if not significantly displaced from the Structure cline; otherwise highlighted).

Finally, we conduct geographical cline analysis. Because the Marmara Sea causes a north‐south division in the region where the two species meet, we arrange localities along two transects with a shared eastern section, running across the Bosphorus in the north and through the hybrid zone in the south (Fig. 4; Fig. S1; Table S3). The clines for Structure *Q* scores, that is the overall species turnover based on all 49 nuclear markers, show a sharp transition between the two species (with cline widths of 9.4 and 39.3 km along the northern and southern transect; 95% confidence intervals of 0.4–15.6 and 27.0–58.7 km). For most (nearly) diagnostic markers, the cline centers do not deviate significantly from that of the Structure clines. However, along the northern and southern transect three and four nuclear markers, involving five nuclear markers in total, have their cline centers significantly displaced eastwards (as does mtDNA), while not a single marker shows a significant westward displacement. Under neutrality, the consistent eastwards displacement of five nuclear markers in the same direction as the mtDNA displacement would be unlikely by chance (0.5^5^ = 0.03).

Under the hypothesis of zone movement, we expect that introgressed alleles would behave neutrally once they had been left behind and so would have wide clines that may explain only a limited proportion of among‐population variation. Some displaced clines have unexpectedly narrow estimated widths but with wide confidence intervals (*amot*, *slc25*; Table S3). Allele frequencies in the east vary widely around the clinal expectations for these loci (Fig. S1) and this sometimes results in models with asymmetric tails having better fits than simple cline models (Table S3). For all loci with displaced centers, tails are to the right (east) as expected. We found a significant excess of residual variation around the cline fits, relative to the binomial expectation, in eight out of 19 markers in one or both transects. While 3/6 displaced loci show excess residual variance in one transect or the other, this was not significantly greater than the 5/13 nondisplaced loci ($\chi_{1}^{2}=\;0.22$ ;Table S4). Furthermore, these departures appear to be driven by individual localities with high residuals, rather than variation among localities in general (Fig. S1). All of the outlier localities are positioned east of the hybrid zone, in the hypothesized species displacement zone. Such outliers are not unexpected in the wake of a moving hybrid zone and can be explained as islands of western alleles, left behind in the eastern species’ range (Macholán et al. 2011). Crucially, these outlier localities do not influence the eastward displacement of the cline fits, although they do tend to make the cline slope shallower to the east of the center.

The abrupt geographical transition between overall parental genotypes is consistent with a tension zone model, where hybridization persists over time as parentals move into the hybrid zone, while introgression is limited by negative selection against hybrids, allowing species to maintain their overall genetic integrity (Barton and Hewitt 1985). The steeper cline in the northern transect (9.4 km vs 39.3 km along the southern transect; Table S3) is probably due to the barrier created by the Bosphorus. Therefore, we estimated lifetime dispersal from linkage disequilibrium in the southern transect, using the hybrid index approach of Barton and Gale (1993) (Table S5). The estimate of 3.66 km/generation (95% confidence interval 0.13–5.90, compatible with observational data of c. 1 km/year in another crested newt species, *T. cristatus* (Arntzen and Wallis 1991; Kupfer and Kneitz 2000)), leads to an expected cline width from neutral diffusion over the ∼12,000 years since contact, assuming a generation time of 3.5 years, of c. 443 km, much wider than the observed cline width (14.8 and 56.5 km along the northern and the southern transect, with 95% confidence intervals of 1.2–24.2 and 18.3–82.6 km; Table S5). The effective selection per locus, based on a model of selection against heterozygotes and inferred from the width of a geographical cline fitted to the hybrid index for nondisplaced markers, is estimated to be 0.011 (0.004–0.019); consistent with estimates from other tension zones where linkage disequilibrium is low (Baldassarre et al. 2014; Hollander et al. 2015).

The strong geographical asymmetry in introgression, from the western toward the eastern species, is consistent with the predictions under westward hybrid zone movement. The markers that show introgression vary in the extent of replacement of local alleles by introgressed ones, but the displacements of cline centers are large compared with the estimated dispersal distance and the width of the hybrid zone (as indicated by the Structure *Q*‐score cline). The intensity of introgression decreases eastwards and not all markers have displaced cline centers along both the northern and southern transect. Considering that the introgression potential of individual selectively neutral alleles increases with their genetic distance from negatively selected alleles (Barton 2000), it is to be expected that recombination has disassociated alleles from their genomic background for only a subset of the studied nuclear markers, while the strongly displaced mtDNA cline is consistent with a complete lack of linkage to the nuclear genome. Furthermore, a moving hybrid zone is expected to accumulate foreign alleles along the way, independently when the zone became split into two by the Marmara Sea, because an element of chance is involved in separating alleles from the advancing wave of species expansion. The chance effects are expected to be greater if the expansion is “pulled” by high fitness of long‐distance migrants into the territory of the replaced species than if it is “pushed” by an advancing wave (Hallatschek et al. 2007; Excoffier et al. 2009), but they always exist.

Asymmetric introgression across a stable hybrid zone could result from (1) prezygotic effects, namely asymmetric hybridization due to sex‐biased dispersal (Petit and Excoffier 2009) or asymmetric mate choice (While et al. 2015), or (2) postzygotic effects, namely biased survival of the sexes (Haldane 1922) or reciprocal‐cross hybrids (Arntzen et al. 2009). While prezygotic effects are not known to occur in *Triturus* newts (Arntzen 2003), postzygotic effects do operate in the hybrid zone between the relatively diverged crested and marbled newts (Arntzen et al. 2009). However, such asymmetries should only be expressed in markers with (1) a sex‐biased transmission, and such markers would disperse under neutral diffusion, or (2) linked to a successful phenotype, and such markers would expand under positive selection. Such explanations, based on asymmetric selection on individual loci, are expected to produce introgression only in a few genome regions and with no necessary consistency in direction. Favoured alleles may spread throughout the introgressed range or they may lose their selective advantage once beyond the main region of admixture, depending on the form of selection. Yet, we observe introgression for markers spread randomly across the genome, over a vast geographical area. This is expected under hybrid zone movement, but highly unlikely under other forms of asymmetric introgression.

## Conclusion

We have tested the hypothesis that a crested newt hybrid zone moved over a considerable time and distance, by documenting unidirectional interspecific introgression for multiple genes. Despite employing a moderately sized dataset we obtain sufficient statistical power. Using three distinct approaches, consulting the geography of genetic admixture, the distribution of species‐diagnostic alleles, and the displacement of geographical clines in relation to the hybrid zone, we confirm a pronounced asymmetry in introgression. Our findings are in line with predictions based on theoretical principles (Barton and Hewitt 1985) and data simulation (Currat et al. 2008), for a genomic footprint left by a receding species, in the region where it was displaced by an expanding one. Hence, without relying on direct observations of hybrid zone movement (and without requiring a detailed understanding of the species’ biology), we provide empirical evidence consistent with hybrid zone movement over an extended period of time.

The crested newt case illustrates that hybridizing species can remain distinct in the face of gene flow, while undergoing substantial range changes, over extended periods of time. Incorporating the spatio‐temporal dynamics of hybrid zones is an important extension of historical biogeography (Hewitt 2011; Taylor et al. 2015). We anticipate that future research will show hybrid zones to be far more mobile over time than is currently appreciated. Moving hybrid zones can make an important contribution to speciation research because, with the introgression of the selectively neutral part of the genome inflated, the contrast of the genomic regions responsible for maintaining species integrity is enhanced, hence facilitating their detection (Currat et al. 2008).

## Supporting information


**Supplementary Methods**.

**Figure S1**. Results of the geographical cline analyses for individual markers and transects. Results for the northern and southern transect are in the left and right column (see Fig. 3 for details). Clines are shown from top to bottom for Structure *Q* score, mtDNA haplotype frequency, and allele frequency for the 12 diagnostic (*amot* ‐ *taf8*) and six near diagnostic markers (*chic* ‐ *wiz*). For each cline model the 95% credible cline region is shown in grey and the sampling sites are denoted as black dots (see Supplementary Table 3 for details).

**Table S1**. Sampling details.
**Table S2**. GenBank Accesion Numbers for mtDNA haplotypes.
**Table S3**. Best fitting geographical cline models selected for the Structure *Q* score and the individual mtDNA and nuclear diagnostic (*amot* ‐ *taf8*) and semi‐diagnostic (*chic* ‐ *wiz*) markers.
**Table S4**. Test whether there is more variation unaccounted for by the cline fit than one would expect given the binomial error model and the sample sizes.
**Table S5**. Estimating dispersal and selection from linkage disequilibrium.
