# Supplementary material for: A genomic footprint of hybrid zone movement in crested newts
Source: Evol Lett. 2017 May 9;1(2):93–101. doi: 10.1002/evl3.9 (PMC6121819; doi:10.1002/evl3.9)
Supplement: Supplementary file 1 — Supplementary Methods. [file EVL3-1-93-s001.docx]

**Supplementary Methods**

**Sampling.** We study two crested newt species: the eastern *Triturus anatolicus* and the western *T. ivanbureschi*. We sampled 428 crested newt individuals from 67 localities (Fig. 1; Supplementary Table 1). For each of the two species we selected 15 reference individuals (three individuals from five localities spread throughout the range, but positioned away from the putative hybrid zone and the ranges of congeneric species) to determine 1) a threshold for Bayesian clustering analysis, and 2) whether individual markers are diagnostic (see below).

**MtDNA sequencing and analysis.** We sequenced 658 bp of an mtDNA gene (ND4) using the protocol described in [Wielstra *et al.* (2013b](#_ENREF_29)) for 378 individuals. For the 50 individuals for which we could not obtain the 658-bp fragment (presumably due to DNA degradation), we sequenced an internal 110-bp fragment using primers TivaIF (5’- ATTTGCATGCGACAAACARA -3’) and TivaIR (5’-TGATGAGATAAGCCCRTGTG-3’). We aligned the 658-bp sequences with the haplotype database of [Wielstra *et al.* (2013b](#_ENREF_29)) and collapsed sequences into haplotypes with MacClade 4.08 ([Maddison & Maddison, 2005](#_ENREF_14)) to determine to which, if any, previously identified haplotypes they belonged (Supplementary Table 2). New haplotypes were assigned to species by constructing a Bayesian phylogeny (Fig. 2) with MrBayes 3.2.2 ([Ronquist *et al.*, 2012](#_ENREF_19)), run on the Cipres Science Gateway ([Miller *et al.*, 2010](#_ENREF_15)). The best fitting model of sequence evolution (GTR+I+G) was determined based on the Akaike information criterion in jModelTest 2.1.7 ([Darriba *et al.*, 2012](#_ENREF_3)). We conducted two, four chain Markov Chain Monte Carlo runs with a temperature of 0.2, a sampling frequency of 0.001 and a burn-in of 25%. Tracer 1.6 ([Rambaut & Drummond, 2013](#_ENREF_18)) was used to confirm stabilization within and convergence between runs (all ESS values > 200). We used the other seven *Triturus* species and *Calotriton asper* as outgroup (data from [Wielstra *et al.* (2010](#_ENREF_27))). We then aligned the 110-bp sequences with these haplotypes and trimmed the set of sequences accordingly. By removing redundancy in MacClade we could determine unambiguously whether sequences belonged to the eastern or western mtDNA type. We determined the average number of nucleotide substitutions (D_XY_) between the two species with DNAsp 5 ([Librado & Rozas, 2009](#_ENREF_12)) for the 658-bp alignment.

**Nuclear DNA sequencing.** For all 428 individuals we sequenced 49 nuclear markers following the protocol presented by [Wielstra *et al.* (2014](#_ENREF_28)) (an additional three markers were excluded because fewer than 20 reads were available for > 20% of the individuals). In brief, we amplified markers of c. 140 bp in length, designed from transcriptome data and positioned in the 3’ untranslated regions of protein coding genes, in five multiplex PCRs. We pooled the multiplexes for each individual and ligated unique tags. Amplicons were sequenced on the Ion Torrent next-generation sequencing platform. The output was processed with a bioinformatics pipeline. First, poor quality reads were filtered out by removing those with a length less than 100 bp and an average quality of less than Q20. Next, reads for each individual were mapped against the targeted nuclear markers using BWA v0.7.3 ([Li & Durbin, 2009](#_ENREF_10)) and SNP/InDel calling was performed with SAMtools v0.1.18 ([Li *et al.*, 2009](#_ENREF_11)). A SAMtools quality score over Q60 was required for SNP/InDels to be retained. Subsequently, alleles were reconstructed by determining the combination of SNPs and InDels in the reads of marker-individual combinations. Marker-individual combinations were required to have at least ten reads, alleles were required to be present in at least 25% of the reads, and a maximum of two alleles were allowed to be present, otherwise a marker-individual combination was considered failed. Finally data was converted into a genotypic data format by recoding the different allelic variants for each marker to a unique integer, resulting in two integers per individual per marker. For the 49 markers, 98.4% of marker-individual combinations had ≥ 20 reads, with a mean coverage of 820.6 (range 0-17,925). We used GENEPOP 4.3 ([Rousset, 2008](#_ENREF_20)) to confirm that there were no significant departures from linkage equilibrium after correcting for multiple testing using the false discovery rate ([Storey, 2002](#_ENREF_21)) approach (http://www.sdmproject.com/utilities/). We observed deviations from Hardy–Weinberg equilibrium for 6 out of 49 markers in 1 to 19 out of 67 localities, after false discovery rate correction. Therefore, we also ran downstream analyses excluding these markers to confirm they did not drive the observed genetic structure (results on Dryad only).

**Bayesian clustering analysis.** We used Structure 2.3.3 ([Pritchard *et al.*, 2000](#_ENREF_16)) to estimate for each individual the fraction of ancestry derived from each of *k* gene pools. We varied *k* over a 1-20 range, with ten replicates per *k* value, and used the admixture model in combination with the correlated allele frequency model, with 1,000,000 iterations after 250,000 iterations of burn-in. In line with the two-species hypothesis, *k* = 2 was the preferred value under the Δ*k* criterion ([Evanno *et al.*, 2005](#_ENREF_6)) as implemented in CLUMPAK ([Kopelman *et al.*, 2015](#_ENREF_8)). Structure *Q* scores are in Supplementary Table 1. For individuals to be assigned to one of the two parental species we set a *Q* score ≥ 0.987 as a threshold, based on the lowest score with which a reference individual was allocated to its respective species. Individuals with a score below this threshold were considered genetically admixed, and those with a *Q* score arbitrarily ≤ 0.850 to either of the two species regarded as considerably admixed.

**Genetic differentiation and population tree.** Divergence between the two species as reflected by their multilocus pairwise F_ST_ value was calculated in GENEPOP ([Rousset, 2008](#_ENREF_20)). We constructed a population tree from allele frequency data with POPTREE 2 ([Takezaki *et al.*, 2010](#_ENREF_22)) based on corrected *F*_ST_ distances, using neighbour-joining with 1000 bootstrap replicates. We excluded the 62 individuals (including six entire localities) highlighted as genetically admixed in Structure to minimize the confounding effect of interspecific gene flow. As POPTREE does not allow markers to be invariant or have no data for an entire locality, we excluded one monomorphic marker, one marker with data missing in at least three localities and six localities (14 individuals) with data missing for at least one other marker.

**Estimating heterozygosity and ancestry.** We focused on a subset of 1) 12 fully diagnostic markers, exhibiting fixed allelic differences between the reference individuals representing the two species, and 2) an additional six nearly-diagnostic markers showing a frequency difference > 0.80 ([Baldassarre *et al.*, 2014](#_ENREF_1); [Larson *et al.*, 2014](#_ENREF_9)). We determined the genomic composition based on heterozygosity (the fraction of markers heterozygous for alleles from each parental species) and ancestry (the fraction of alleles derived from each parental species) with the R ([R-Development-Core-Team, 2016](#_ENREF_17)) package HIest ([Fitzpatrick, 2012](#_ENREF_7)). Marker-individual combinations for which either or both alleles were not present in the reference individuals (and hence for which evolutionary origin could not be established) were treated as missing data. We required individuals to have information available for at least nine (75%) of the 12 diagnostic markers, leading us to exclude 30 individuals. Data (see Supplementary Table 1) were plotted (Fig. 3) in STATISTICA 7 (www.statsoft.com).

**Geographical cline analysis.** We fitted geographical clines with the R package HZAR ([Derryberry *et al.*, 2014](#_ENREF_4)) to 1) the Structure *Q* score based on all 49 nuclear markers, 2) the mtDNA marker, and 3) each of the 12 diagnostic and six nearly-diagnostic nuclear markers. We tested all 15 models, estimating cline centre and width, and combining three possible fits of allele frequencies at the cline ends (fixed to 0 and 1, observed values or estimated values) with five possible tail fits (none fitted, left only, right only, mirrored or both estimated separately), and used the lowest Akaike information criterion score corrected for small sample size (AICc) to select the best fitting one. Western species locality 15 and eastern species locality 66 were considered as the starting and end point of clines, while 35 intermediary localities with a sample size *n* ≥ 3 were arranged into two transects that differed in their western section: one running north of the Marmara Sea, across the Bosphorus, and one running south of the Marmara Sea, across the hybrid zone (Fig. 4; Supplementary Fig. 1). To determine a locality’s position in kilometres along a transect we used its perpendicular from an imaginary west-east line with a latitude of 40.78°N (the mean latitude of all included localities). For each marker, individuals with missing data or alleles of uncertain evolutionary origin were excluded. Marker clines were considered to be significantly displaced if 1) the two log-likelihood unit support limits for the cline centre did not overlap with those for the Structure cline, and 2) a model with the cline centre refitted to that of the Structure cline produced an AICc score at least two points higher ([Baldassarre *et al.*, 2014](#_ENREF_1); [While *et al.*, 2015](#_ENREF_25)) (Supplementary Table 3). We confirmed that cline displacement was not appreciably affected by variation in the tails fitted by repeating analyses without tails (results on Dryad only). Since HZAR does not allow inclusion of variation in allele frequencies among local populations in its cline models, we tested for residual variation that exceeded the expected binomial variance. Using the best fitting model for each locus and transect, we calculated the expected allele frequency at each sample location and used glm in the R package lme4, with binomial distribution, to calculate the residual variation in allele count. The residual deviance was tested against a χ2-distribution, with the residual degrees of freedom, which is the expectation if the binomial model is adequate (Supplementary Table 4).

**Estimating selection.** We followed the approach outlined by [Barton and Gale (1993](#_ENREF_2)) to estimate effective selection, using life-time dispersal distance inferred from admixture linkage disequilibrium (LD) from the hybrid index, and comparing the observed cline width with that expected under neutrality (Supplementary Table 5). For each transect we determined the average hybrid index per locality, based on those (nearly) diagnostic nuclear markers that are not significantly displaced, and fitted clines in HZAR. The average LD was determined for each locality from the variance in the hybrid index and the allele frequency across markers (equation 2b in [Barton and Gale (1993](#_ENREF_2))). We regressed LD on the product of the average western*eastern allele frequencies across loci at each locality and estimated LD at the theoretical centre of the hybrid zone (where $\bar{pq} = 0.25$). Lifetime dispersal distance per generation, *σ*, was estimated, while taking into account that our total sample of individuals (*n*) was a mix of pre-metamorphic individuals (*n_pre_*), that did not yet have the opportunity to disperse, and post-metamorphic individuals (*n_post_*), that did, as $\sigma=\frac{n\text{pre}}{n}*\sqrt{rD\omega\text{o}\text{2}}+\frac{n\text{post}}{n}*\sqrt{rD\omega\text{o}\text{2}/(1+r)}$. The recombination rate, *r*, was estimated as 0.431 following equation 6 in [Macholán *et al.* (2007](#_ENREF_13)), considering that *Triturus* has 12 chromosomes, using 27.5 chiasmata based on the average observed in another crested newt species, *T. carnifex* ([Wallace *et al.*, 1997](#_ENREF_24)), and setting r_0_ to 0.001, *D* was the estimated LD at the cline centre and *ω_o_* the observed cline width. The expected cline width under neutral diffusion was determined as $\omega\text{n} = 2.51\sigma\surd t$, where *t* is the number of generations since secondary contact ([Barton & Gale, 1993](#_ENREF_2)). As the age of maturity for both species is 3-4 years ([Üzüm, 2006](#_ENREF_23)), we set generation time to 3.5 years. Secondary contact is thought to have become established upon the closing of the Izmit Gulf – Lake Sapanca – Sakarya Valley waterway at the onset of the Holocene, c. 12 Ka ([Elmas, 2003](#_ENREF_5); [Wielstra *et al.*, 2013a](#_ENREF_26)). This gives c. 3,429 generations since secondary contact. We determined the effective selection pressure per marker as $s\text{*} = (2\sigma/\omega\text{o})\text{2}$, assuming a model of selection against heterozygotes ([Barton & Gale, 1993](#_ENREF_2)).

**References**

Baldassarre, D.T., White, T.A., Karubian, J. & Webster, M.S. (2014) Genomic and morphological analysis of a semipermeable avian hybrid zone suggests asymmetrical introgression of a sexual signal. *Evolution*, **68**, 2644-2657.

Barton, N.H. & Gale, K.S. (1993) Genetic analysis of hybrid zones. *Hybrid zones and the evolutionary process* (ed. by R.G. Harrison), pp. 13-45. Oxford University Press, New York.

Darriba, D., Taboada, G.L., Doallo, R. & Posada, D. (2012) jModelTest 2: more models, new heuristics and parallel computing. *Nat Meth*, **9**, 772-772.

Derryberry, E.P., Derryberry, G.E., Maley, J.M. & Brumfield, R.T. (2014) HZAR: hybrid zone analysis using an R software package. *Mol. Ecol. Resour.*, **14**, 652-663.

Elmas, A. (2003) Late Cenozoic tectonics and stratigraphy of northwestern Anatolia: the effects of the North Anatolian Fault to the region. *Int. J. Earth Sci.*, **92**, 380-396.

Evanno, G., Regnaut, S. & Goudet, J. (2005) Detecting the number of clusters of individuals using the software structure: a simulation study. *Mol. Ecol.*, **14**, 2611-2620.

Fitzpatrick, B.M. (2012) Estimating ancestry and heterozygosity of hybrids using molecular markers. *BMC Evol. Biol.*, **12**, 131.

Kopelman, N.M., Mayzel, J., Jakobsson, M., Rosenberg, N.A. & Mayrose, I. (2015) Clumpak: a program for identifying clustering modes and packaging population structure inferences across K. *Mol. Ecol. Resour.*, **15**, 1179–1191.

Larson, E.L., White, T.A., Ross, C.L. & Harrison, R.G. (2014) Gene flow and the maintenance of species boundaries. *Mol. Ecol.*, **23**, 1668-1678.

Li, H. & Durbin, R. (2009) Fast and accurate short read alignment with Burrows–Wheeler transform. *Bioinformatics*, **25**, 1754-1760.

Li, H., Handsaker, B., Wysoker, A., Fennell, T., Ruan, J., Homer, N.*, et al.* (2009) The Sequence Alignment/Map format and SAMtools. *Bioinformatics*, **25**, 2078-2079.

Librado, P. & Rozas, J. (2009) DnaSP v5: a software for comprehensive analysis of DNA polymorphism data. *Bioinformatics*, **25**, 1451-1452.

Macholán, M., Munclinger, P., Šugerková, M., Dufková, P., Bímová, B., Božíková, E.*, et al.* (2007) Genetic analysis of autosomal and X-linked markers across a mouse hybrid zone. *Evolution*, **61**, 746-771.

Maddison, D.R. & Maddison, W.P. (2005) *MacClade: Analysis of Phylogeny and Character Evolution*. Sinauer Associates, Sunderland, MA.

Miller, M.A., Pfeiffer, W. & Schwartz, T. (2010) Creating the CIPRES Science Gateway for inference of large phylogenetic trees. In: *Proceedings of the Gateway Computing Environments Workshop (GCE)*, pp. 1-8, New Orleans, LA.

Pritchard, J.K., Stephens, M. & Donnelly, P. (2000) Inference of population structure using multilocus genotype data. *Genetics*, **155**, 945-959.

R-Development-Core-Team (2016) *R: A language and environment for statistical computing*. R Foundation for Statistical Computing, Vienna, Austria.

Rambaut, A. & Drummond, A.J. (2013) *Tracer v1.6, Available from* [*http://tree.bio.ed.ac.uk/software/tracer/*](http://tree.bio.ed.ac.uk/software/tracer/).

Ronquist, F., Teslenko, M., van der Mark, P., Ayres, D.L., Darling, A., Höhna, S.*, et al.* (2012) MrBayes 3.2: Efficient Bayesian phylogenetic inference and model choice across a large model space. *Syst. Biol.*,

Rousset, F. (2008) genepop'007: a complete re-implementation of the genepop software for Windows and Linux. *Mol. Ecol. Resour.*, **8**, 103-106.

Storey, J.D. (2002) A direct approach to false discovery rates. *J. Roy. Stat. Soc. B Met.*, **64**, 479-498.

Takezaki, N., Nei, M. & Tamura, K. (2010) POPTREE2: software for constructing population trees from allele frequency data and computing other population statistics with Windows interface. *Mol. Biol. Evol.*, **27**, 747-752.

Üzüm, N. (2006) *Türkiye'deki Triturus karelinii (Strauch 1870) (Urodela: Salamandridae) populasyonlarında yaş tayini: populasyonların büyüme, yaş ve boy bakımından karşılaştırılması*. Adnan Menderes Üniversitesi, Aydın.

Wallace, H., Wallace, B. & Badawy, G. (1997) Lampbrush chromosomes and chiasmata of sex-reversed crested newts. *Chromosoma*, **106**, 526-533.

While, G.M., Michaelides, S., Heathcote, R.J.P., MacGregor, H.E.A., Zajac, N., Beninde, J.*, et al.* (2015) Sexual selection drives asymmetric introgression in wall lizards. *Ecology Letters*, **18**, 1366-1375.

Wielstra, B., Baird, A.B. & Arntzen, J.W. (2013a) A multimarker phylogeography of crested newts (*Triturus cristatus* superspecies) reveals cryptic species. *Mol. Phylogenet. Evol.*, **67**, 167-175.

Wielstra, B., Espregueira Themudo, G., Güclü, Ö., Olgun, K., Poyarkov, N.A. & Arntzen, J.W. (2010) Cryptic crested newt diversity at the Eurasian transition: the mitochondrial DNA phylogeography of Near Eastern *Triturus* newts. *Mol. Phylogenet. Evol.*, **56**, 888-896.

Wielstra, B., Duijm, E., Lagler, P., Lammers, Y., Meilink, W.R.M., Ziermann, J.M.*, et al.* (2014) Parallel tagged amplicon sequencing of transcriptome-based genetic markers for *Triturus* newts with the Ion Torrent next-generation sequencing platform. *Mol. Ecol. Resour.*, **14**, 1080-1089.

Wielstra, B., Crnobrnja-Isailović, J., Litvinchuk, S.N., Reijnen, B.T., Skidmore, A.K., Sotiropoulis, K.*, et al.* (2013b) Tracing glacial refugia of *Triturus* newts based on mitochondrial DNA phylogeography and species distribution modeling. *Front. Zool.*, **10**, 13.
