# Supplementary material for: A genomic footprint of hybrid zone movement in crested newts
Source: Evol Lett. 2017 May 9;1(2):93–101. doi: 10.1002/evl3.9 (PMC6121819; doi:10.1002/evl3.9)

Supplementary Figures

Supplementary Fig. 1. Results of the geographical cline analyses for individual markers and transects. Results for the northern and southern transect are in the left and right column (see Fig. 3 for details). Clines are shown from top to bottom for Structure *Q* score, mtDNA haplotype frequency, and allele frequency for the 12 diagnostic (*amot* - *taf8*) and six semi-diagnostic markers (*chic* - *wiz*). For each cline model the 95% credible cline region is shown in grey and the sampling sites are denoted as black dots (see Supplementary Table 3 for details).

<u>Northern transect:</u> <u>Southern transect:</u>

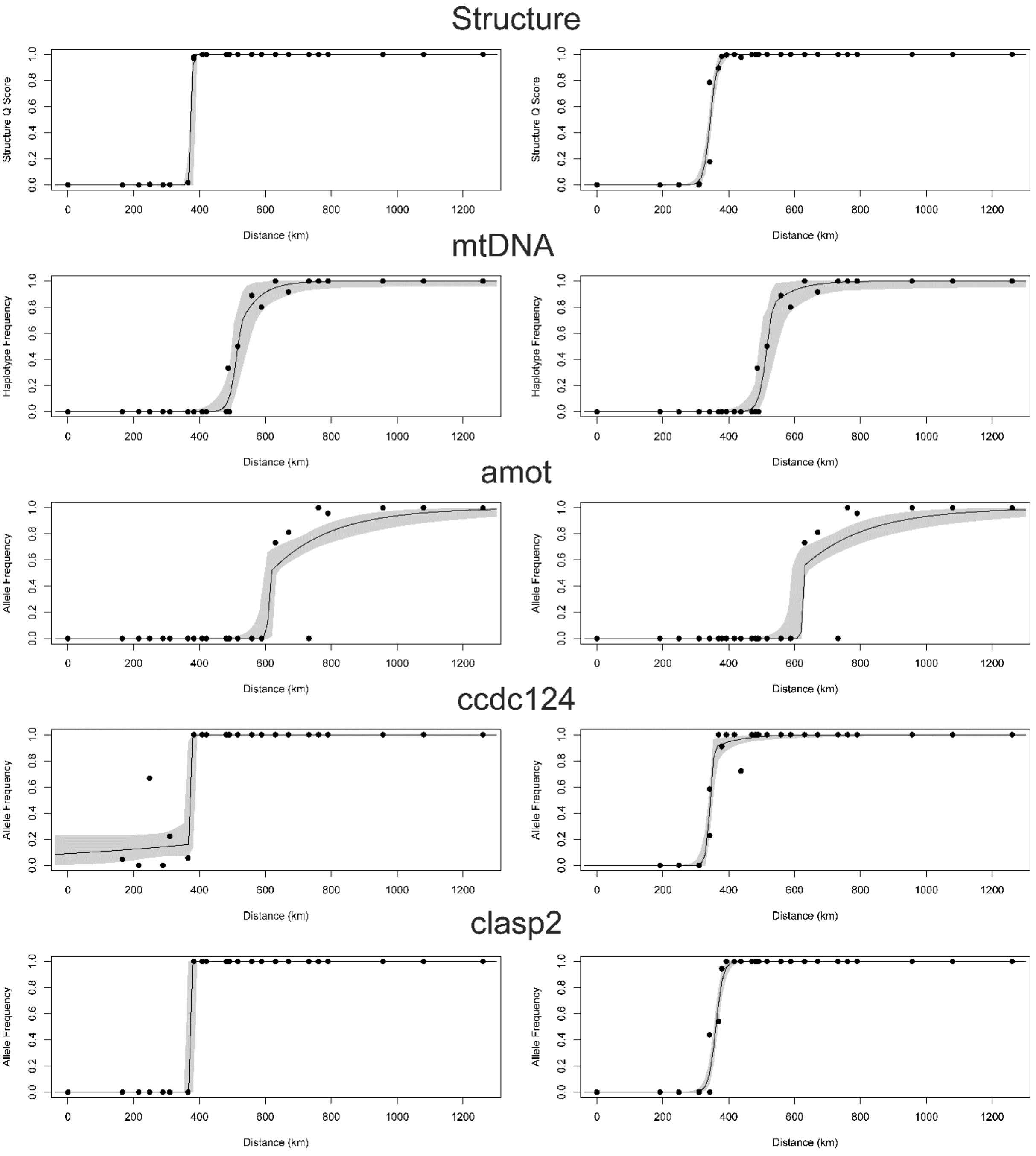

Supplementary Fig. 1. *Continued.*

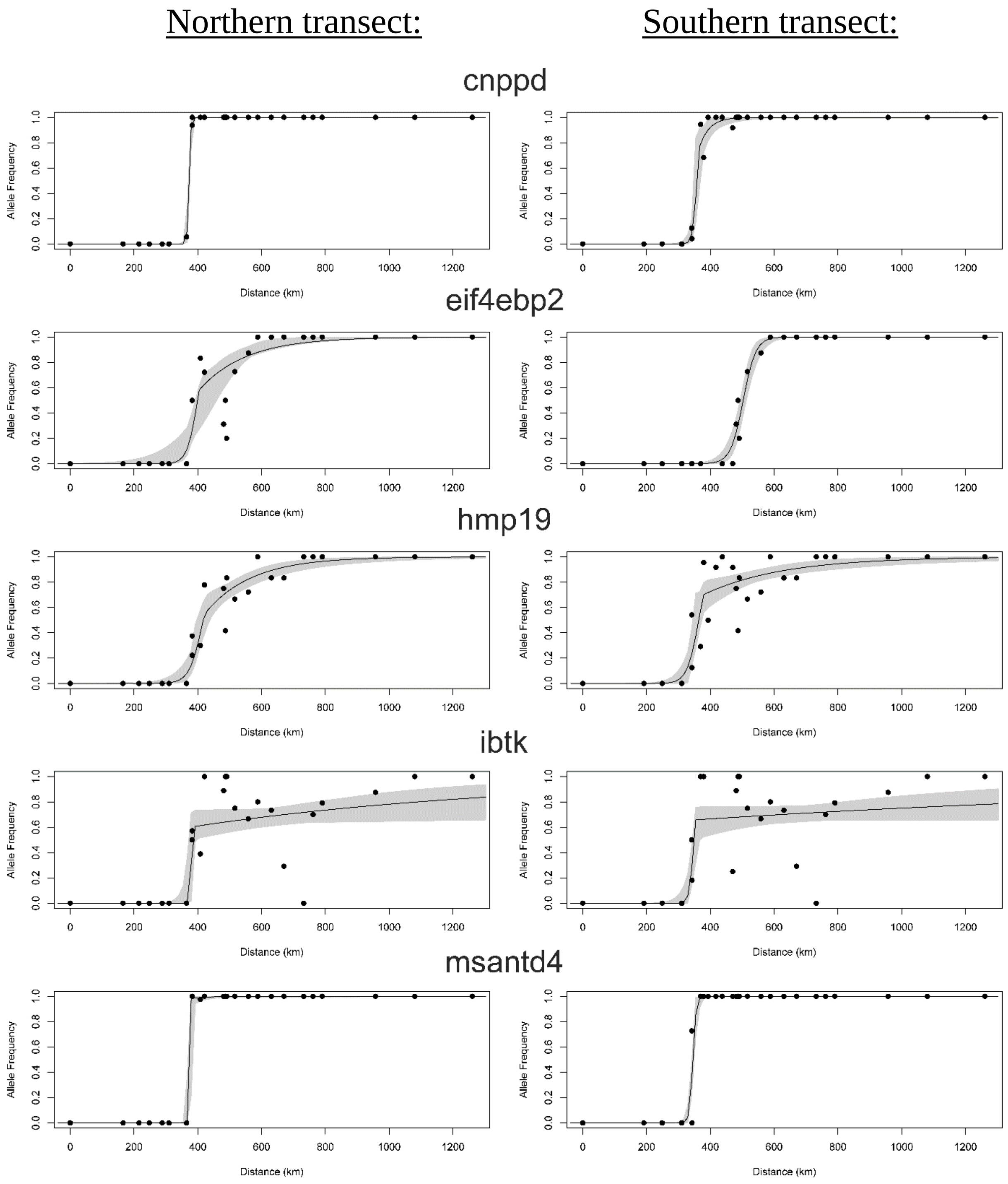

Supplementary Fig. 1. *Continued.*

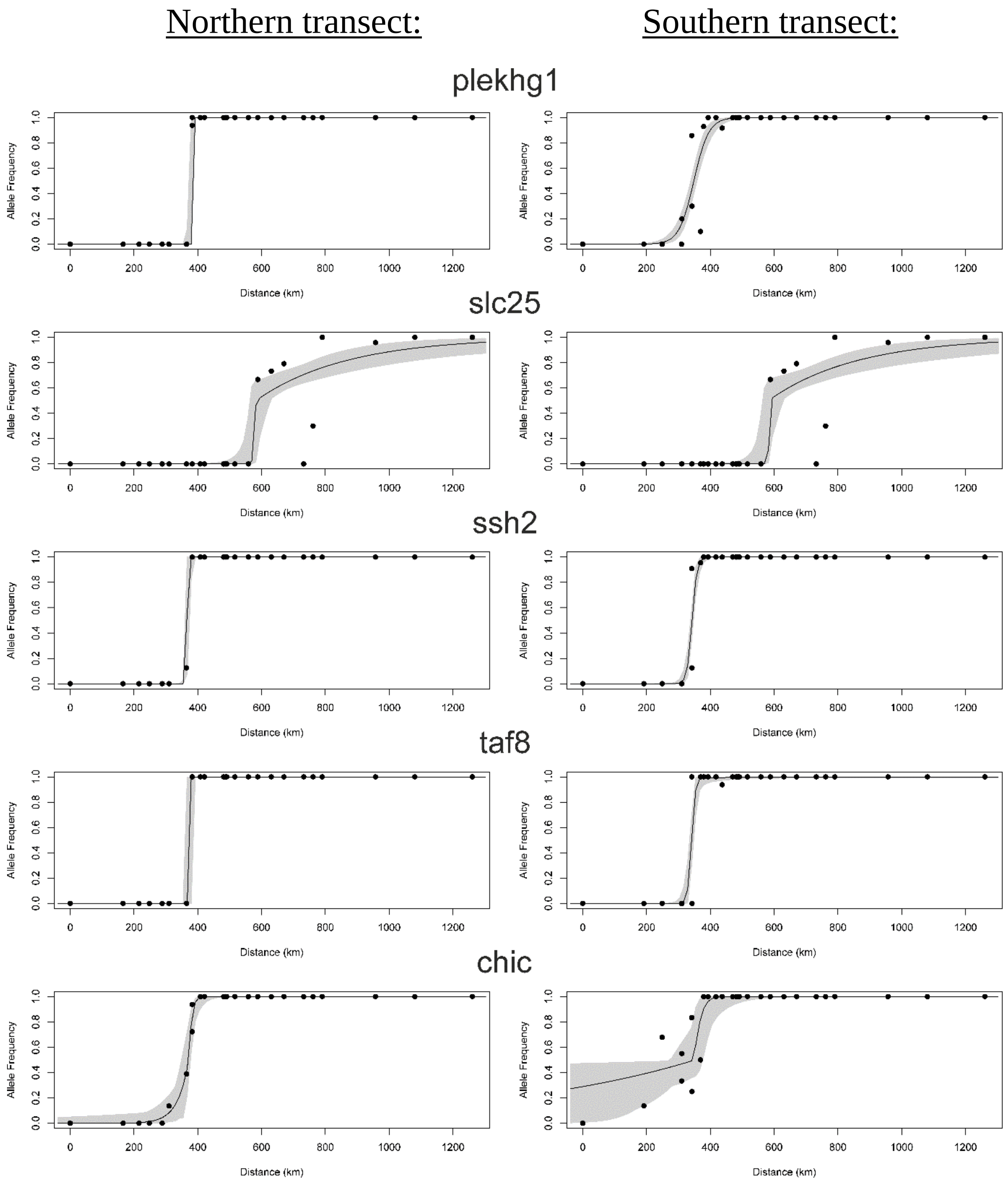

Supplementary Fig. 1. *Continued.*

Northern transect: Southern transect:

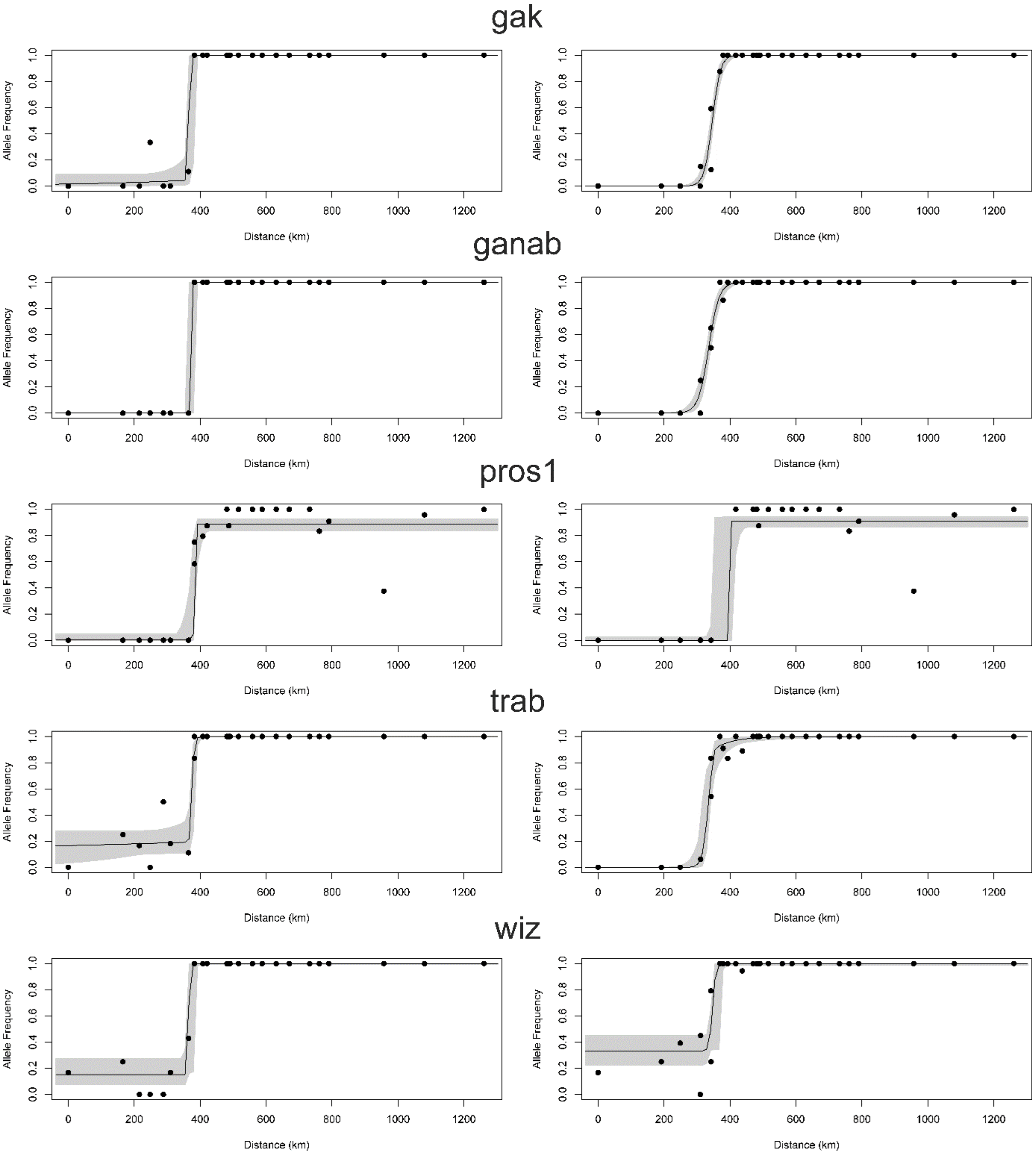

Supplement: Supplementary file 2 — Figure S1. Results of the geographical cline analyses for individual markers and transects. Results for the northern and southern transect are in the left and right column (see Fig. 3 for details). Clines are shown from top to bottom for Structure Q score, mtDNA haplotype frequency, and allele frequency for the 12 diagnostic (amot ‐ taf8) and six near diagnostic markers (chic ‐ wiz). For each cline model the 95% credible cline region is shown in grey and the sampling sites are denoted as black dots (see Supplementary Table 3 for details). [file EVL3-1-93-s002.pdf]
